# Thiopurine *S*-methyltransferase activity in Nigerians: phenotypes and activity reference values

**DOI:** 10.1186/s13104-018-3237-5

**Published:** 2018-02-14

**Authors:** Ayorinde Adehin, Oluseye O. Bolaji

**Affiliations:** 1 Department of Pharmaceutical Chemistry, Faculty of Pharmacy, Obafemi Awolowo University, Ile-Ife, Osun State Nigeria; 2 Institute of Biomedical and Pharmaceutical Sciences, Guangdong University of Technology, Guangzhou, Guangdong Province China

**Keywords:** TPMT, Metabolic phenotypes, Thiopurine toxicity, Nigerians

## Abstract

**Objectives:**

This study assessed the activity of thiopurine *S*-methyltransferase (TPMT) in Nigerians with a view to providing data on susceptibility to thiopurine toxicity, and as well generate reference activity values for clinical use.

**Results:**

TPMT activity, expressed as the amount of 6MMP in ng/mL after 1 h incubation at 37 °C per haemoglobin (U/g Hb), varied between 2.34 and 63.50 U/g Hb in the study population. Poor metabolic phenotypes, characterised by an activity values below 8.41 U/g Hb, were observed in 20% of the study subjects. Intermediate metabolizers had activity values between 8.41 and 16.13 U/g Hb. Fast and very fast metabolizers were characterised by activity values of 16.20–56.22 and > 56.22 U/g Hb, respectively. These findings suggest that a potentially huge discordance between TPMT phenotype and genotype exist in Nigerians, and emphasizes the superiority of a prior determination of TPMT metabolic phenotype in ensuring the safety of thiopurine drug administration in the population.

## Introduction

Thiopurine *S*-methyltransferase (TPMT) is a cytosolic enzyme that S-methylates heterocyclic and aromatic sulfhydryl compounds, and has been found to exhibit varied levels of activity in tissues, a phenomenon largely due to polymorphisms in its gene [1]. Inheritance is autosomal codominant [2] and the various populations studied to date have shown marked interindividual variation in enzyme activity.

TPMT plays a significant role in the biotransformation of clinically important thiopurine drugs used in the management of leukaemia, inflammatory bowel diseases (IBD), rheumatic diseases and dermatological conditions. These drugs are also used as immunosuppressants in the aftermath of organ transplant [3]. Thiopurines are metabolized to generate cytotoxic thioguanine nucleotides (e.g. 6-TGN) whose incorporation into nucleic acid materials form the basis of their immunosuppressive properties [4]. TPMT, however, inactivates thiopurines and functional polymorphisms in its gene can potentially alter the balance of this metabolic pathway. Haematological toxicity or altered antitumor effectiveness are the usual results of the accumulation of cytotoxic thioguanine nucleotides in instances of impaired thiopurine metabolism by TPMT [3, 5].

Genotyping remains an unambiguous method of detecting non-functional *TPMT* alleles with relatively high genotype-to-phenotype correlation of over 67% from some studies [5]. However, some non-genetic factors and genetic variation in the regulatory region of the *TPMT* gene [6] are known to alter phenotypes. This, hence, emphasizes the importance of phenotype determination in the precise classification of the TPMT metabolic status of individuals.

The present study, the first in a Nigerian population, thus aims to give information on the likely TPMT metabolic phenotypes and as well provide clinically useful reference activity values for the routine assessment of susceptibility to thiopurine toxicity.

## Main text

### Methods

One hundred healthy, unrelated adult subjects composed of 87 males and 13 females who were nonsmokers, were recruited. Ninety-three of these volunteers were from the *Yoruba* ethnic group while the other 8 were *Igbo*. The study population had a mean age ± SD of 28.78 ± 3.98 years. All subjects had never been on thiopurine drugs, and were also not on any form of therapy in at least 2 months preceding the study. Each volunteer provided 5 mL of whole blood which was collected in heparinized tubes. Erythrocytes prepared from the collected blood samples were lysed for haemolysates and incubated with 6-mercaptopurine (6MP) in the presence of *S*-adenosylmethionine (SAM) to assess TPMT activity via its production of 6-methylmercaptopurine (6MMP) as earlier described by Oselin et al. [6].

A validated analytical method using a HPLC (1100 series from Agilent Technologies, Palm Alto, USA) device fitted with a quaternary pump and a Diode array UV detector was employed. Samples were injected through a Rheodyne model 7725i valve (Cotati, California; USA) fitted with a 20 μL loop, and analytes were resolved with a reverse phase C18 Zorbax Eclipse XDB column (4 μm, 125 × 4.6 mm i.d.; Agilent technologies, Palm Alto, USA). Separation was achieved with a mobile phase composed of sodium dihydrogen phosphate buffer (0.01 M, pH 2.68) and acetonitrile in a gradient (Table 1) at 27 °C with eluate monitoring at 297 nm.

**Table 1:** Gradient table for the HPLC analysis of 6-methylmercaptopurine

Time (min)	Acetonitrile (%)	Phosphate buffer (%)	Flow rate (mL/min)
0.00	2	98	1.2
2.00	2	98	1.2
2.50	10	90	1.5
6.00	10	90	1.5
6.05	2	98	1.2

Haemoglobin content of the hemolysate was quantified using a colorimetric cyanmethemoglobin method. Haemoglobin-normalized TMPT activity values, computed as the amount of 6MMP in ng/mL after 1 h incubation at 37 °C per haemoglobin (U/g Hb), were subsequently generated. A Rosin–Rammler–Sperling–Weibull (RRSW) distribution analysis [7] was applied to log-transformed normalized values in order to identify breakpoints needed to describe prevalent phenotypic subgroups in the study population. Subgroups were described by the equation: $$F\left( N \right) = 1 - \exp \left[ { - \,{\raise0.7ex\hbox{$X$} \!\mathord{\left/ {\vphantom {X \alpha }}\right.\kern-0pt} \!\lower0.7ex\hbox{$\alpha $}}} \right]^{\beta }$$ where F(N) was the cumulative frequency at a particular log-transformed TPMT activity value ‘X’, while α and β were the scale and slope parameters, respectively. Initial breakpoints were arbitrarily chosen after data inspection, and F(N) adjusted until ‘β’ assumed a Gaussian distribution (adjudged by values between 3.5 and 4.5). The best distribution from several iterations within each subgroup was assessed and chosen based on a goodness of fit analysis using the χ^2^ test.

### Results

**Fig. 1:**
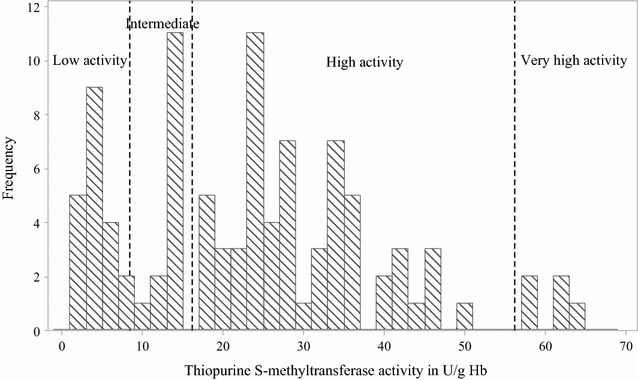
A frequency distribution histogram showing thiopurine *S*-methyltransferase activity, TPMT, activity values for 100 healthy, unrelated Nigerian subjects. U/g Hb: ‘unit per gram haemoglobin’ which is the amount of 6-methylmercaptopurine in ng/mL generated after a 1-h incubation at 37 °C per haemoglobin content

**Fig. 2:**
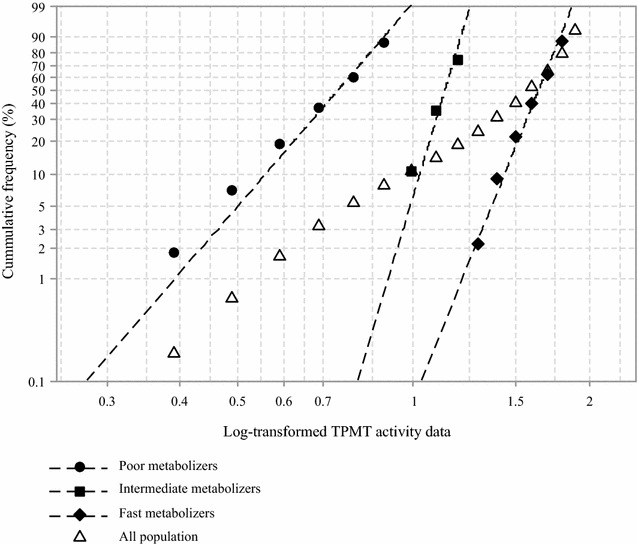
A Weibull plot showing the cumulative frequency distribution of log-transformed thiopurine *S*-methyltransferase activity in 100 healthy Nigerian subjects. Plots of TPMT subgroups’ activity data, and that of the entire population data are shown. TPMT, thiopurine *S*-methyltransferase

All analytes were resolved in 6.5 min with the retention times for 6MP, SAM and 6MMP being 2.4, 4.1 and 5.4 min, respectively. Assay recovery, studied between 5 and 250 ng/mL, was > 83%, while the coefficients of variation for intra- and inter-assay imprecision were < 3%. TPMT activity values varied between 4.88 and 158.85 ng/mL/h, and the corresponding haemoglobin-normalized activity values ranged between 2.34 and 63.50 U/g Hb. Null TPMT activity was recorded in two subjects. The presence of TPMT metabolic phenotypes was evidenced in the preliminary frequency distribution histogram plot of the population data (Fig. 1). Thereafter, two RRSW-functions defining breakpoints at 8.41 and 16.13 U/g Hb were derived after analysis (Fig. 2). By inference, the metabolic phenotypes identified comprised poor (< 8.41 U/g Hb, 20%), intermediate (8.41–16.13 U/g Hb, 14%) and fast (16.20–56.22 U/g Hb, 61%) metabolizers. The Weibull distribution defined by the second RRSW-function, with a breakpoint at 16.13 U/g Hb, only assumed a Gaussian distribution after the exclusion of activity values > 56.23 U/g Hb. This, hence, necessitated the inclusion of a fourth group, observed at a frequency of 5%, comprising individuals with marginally faster TPMT metabolic phenotype.

### Discussion

Genetic polymorphism in the *TPMT* gene with functional consequences has been reported as a major cause of the wide interindividual differences observed in TPMT activity. This has been used to explain variations in the metabolism, toxicity, and therapeutic efficacy of thiopurine drugs. It also known that TPMT activity vary over time in relation to continued administration of thiopurines and patient clinical condition [1, 8]. The present study assessed TPMT metabolic phenotypes in Nigerians and observed a much higher frequency (20%) of poor metabolizers than had been reported in other populations such as Italian-Caucasians (0.1%), Brazilians (0.3%), and German-Caucasians (0.6%) where phenotypes had been predicted using *TPMT* genotypes [9–11].

Interestingly, a recent study in Nigerians [12] which detected only the TPMT*3C allele found just one heterozygous individual in all 180 subjects studied. This would have suggested an estimated 0.6% poor metabolizers, thus emphasizing the pitfall in the prediction of TPMT metabolic phenotypes solely from genetic data for this population. A similar phenotype–genotype disparity has also been reported in Argentines [13] who were identified as intermediate metabolizers from phenotypic data but with only 12 of 21 such individuals being carriers of a copy of the generally assessed defective *TPMT* allele.

While the high frequency of poor metabolizers in the present study may not be explainable by projections of documented genetic data on some *TPMT* alleles in Nigerians, potential reasons may lie in other factors such as promoter polymorphisms [14], variable number of tandem repeats in the promoter region of *TPMT* [15, 16], and new/unstudied mutations in the *TPMT* gene. It is, hence, likely, these other genetic factors highlighted might have more significant roles in the determination of TPMT metabolic phenotypes for this population. This is quite possible because non-genetic modulators of TPMT activity (such as long period of thiopurine use, TPMT inhibitor-drug like as allopurinol, frusemide and naproxen [17, 18] and other unidentified factors) could not have interfered with the in vitro assay of TPMT activity as implemented in the present study.

Since individuals with very low or undetectable levels of TPMT activity are known to develop severe myelosuppression [19] when treated with standard doses of thiopurines, complete reliance on *TPMT* genetic data may not be sufficient, especially among Nigerian users of thiopurine drugs. Moreover, individuals with poor TPMT metabolic phenotypes are preferably not administered thiopurines, and if given, very low doses in the region of less than 10% of the standard doses have been recommended [20]. It is, however, worth stating that although thiopurine toxicity resulting from its altered metabolism has been significantly correlated with TPMT activity, later studies have also identified the roles of inosine triphosphatase (ITPA) [21] and Nudix Hydrolase 15 (NUDT15) [22] in predicting such toxicities.

The present study also identified intermediate metabolizers, with lesser risk of adverse events, for whom thiopurine therapy may be better optimized if properly classified prior to treatment. For example, a study by Gardiner et al. [23] in IBD patients suggested that intermediate TPMT metabolizers might benefit more from a threefold reduction in standard thiopurine doses needed to achieve therapeutic 6-TGN concentrations of ‘> 235 pmol’ per ‘8 × 10^8^ RBCs’. Marginally-higher TPMT activity, seen in some individuals in the present study, had been reported in other populations as well but with no identified genetic/non-genetic causes.

### Conclusion

This study suggests that more Nigerians than projected may be at the risk of thiopurine induced adverse events, and thus underscores the need for proper phenotypic classification of recipients of clinically relevant TPMT-substrate drugs prior to commencement of therapy.

## Limitations

A limitation of the present study is the absence of genetic data on the major functional *TPMT* variants that are prevalent in Africans. This, in addition to data on polymorphisms in the *TPMT* gene promoter region and/or variable number of tandem repeats, would have provided more insight into the likely reasons for the high number of poor metabolizers observed in the population.

## Abbreviations

TPMT: thiopurine *S*-methyltransferase
IBD: inflammatory bowel disease
6-TGN: 6-thioguanine nucleotide
6MP: 6-mercaptopurine
SAM: *S*-adenosylmethionine
6MMP: 6-methylmercaptopurine
RRSW: Rosin–Rammler–Sperling–Weibull
ITPA: inosine triphosphatase
NUDT15: Nudix Hydrolase 15
RBC: red blood cells
